# The Synergic Immunomodulatory Effect of Vitamin D and Chickpea Protein Hydrolysate in THP-1 Cells: An In Vitro Approach

**DOI:** 10.3390/ijms252312628

**Published:** 2024-11-25

**Authors:** Ángela Alcalá-Santiago, Rocío Toscano-Sánchez, José Carlos Márquez-López, José Antonio González-Jurado, María-Soledad Fernández-Pachón, Belén García-Villanova, Justo Pedroche, Noelia María Rodríguez-Martín

**Affiliations:** 1 Department of Nutrition and Food Science, Faculty of Pharmacy, University of Granada, 18071 Granada, Spain; angela.alcala@ugr.es (Á.A.-S.); belenv@ugr.es (B.G.-V.); 2 Instituto de Investigación Biosanitaria ibs. GRANADA, 18012 Granada, Spain; 3 Department of Medical Biochemistry, Molecular Biology, and Immunology, Faculty of Medicine, University of Seville, Av. Dr. Fedriani 3, 41071 Seville, Spain; mtoscanos@us.es; 4 Group of Plant Proteins, Instituto de la Grasa-CSIC, 41013 Seville, Spain; jcmarquez@ig.csic.es (J.C.M.-L.); nmrodriguez@ig.csic.es (N.M.R.-M.); 5 Physical and Sport Education, Physical Performance and Sports Research Center, University Pablo de Olavide, 41013 Sevilla, Spain; jagonjur@upo.es; 6 Area of Nutrition and Food Sciences, Department of Molecular Biology, and Biochemistry Engineering, University Pablo de Olavide, 41013 Seville, Spain; msferpac@upo.es

**Keywords:** vitamin D, protein hydrolysate, synergistic interaction, NF-κB, bioactive compounds, anti-inflammatory

## Abstract

Vitamin D (VD), a crucial micronutrient, regulates bone health and immune responses. Recent studies suggest that VD may confer protective effects against chronic inflammatory diseases. Additionally, plant-based peptides can show biological activities. Furthermore, the supplementation of protein hydrolysates with VD could potentially enhance the bioactivity of peptides, leading to synergistic effects. In this study, THP-1 cells were exposed to low concentrations of Lipopolysaccharide (LPS) to induce inflammation, followed by treatment with vitamin D at different concentrations (10, 25, or 50 nM) or a chickpea protein hydrolysate (“H30BIO”) supplemented with VD. The cytotoxicity of VD was evaluated using viability assay to confirm its safety. The cytokine secretion of TNF-α, IL-1β, and IL6 was assessed in the cell supernatant, and the gene expression of *TNF-α*, *IL-1β*, *IL6*, *IL8*, *CASP-1*, *COX2*, *NRF2*, *NF-ĸB*, *NLRP3*, *CCL2*, *CCR2*, *IP10*, *IL10*, and *RANTES* was quantified by qRT-PCR. Treatment with VD alone significantly decreased the expression of the pro-inflammatory genes *TNF-α* and *IL6*, as well as their corresponding cytokine levels in the supernatants. While *IL-1β* gene expression remained unchanged, a reduction in its cytokine release was observed upon VD treatment. No dose-dependent effects were observed. Interestingly, the combination of VD with H30BIO led to an increase in *TNF-α* expression and secretion in contrast with the LPS control, coupled with a decrease in *IL-1β* levels. Additionally, genes such as *IP10*, *NF-κB*, *CCL2*, *COX2*, *NRF2*, and *CASP-1* exhibited notable modulation, suggesting that the combination treatment primarily downregulates NF-κB-related gene activity. This study demonstrates a synergistic interaction between VD and H30BIO, suggesting that this combination may enhance pathways involving TNF-α, potentially aiding in the resolution and modulation of inflammation through adaptive processes. These findings open new avenues for research into the therapeutic applications of enriched protein hydrolysates with VD to manage low-grade inflammatory-related conditions.

## 1. Introduction

VD is a fat-soluble vitamin with critical functions in human physiology. This vitamin exists in two forms: D3 (cholecalciferol) and D2 (ergocalciferol). The most well-known function of VD is bone mineralization, achieved by regulating the levels of calcium and phosphorus in the bone matrix [1,2]. A significant aspect supporting the role of VD in immune regulation is the expression of the VD receptor (VDR) on most immune cells, including B and T lymphocytes, monocytes, macrophages, and dendritic cells. Furthermore, VD can be locally metabolized by immune cells, converting from 25-hydroxyvitamin D3 (25(OH)D3) to its active form 1,25-dihydroxyvitamin D3 (1,25(OH)2D3) [2,3].The combined action of VD and the nuclear receptor VDR exert a modulating effect on the immune response by promoting the differentiation of dendritic cells and regulatory T cells, while reducing Th17 helper T cell responses and the secretion of inflammatory cytokines [2]. Additionally, VD has the ability to suppress adaptive immunity and modulate inflammation, making it a potential target for therapeutic interventions [4,5,6]. Experimental models have demonstrated that VD downregulates T helper 1 (Th1)-mediated immune responses, inhibiting the production of pro-inflammatory cytokines such as interferon gamma (IFN-γ), Interleukin 6 (IL6), Interleukin 2 (IL2), and tumor necrosis factor alpha (TNF-α) [2,7]. The immunomodulatory activity of VD also enhances Th2 cell activity and regulatory T cells (Treg) [8]. In vitro assays show that VD3 promotes dendritic cell differentiation and high levels of anti-inflammatory cytokines (e.g., Interleukin 10 (IL10)), as well as the inhibition of pro-inflammatory cytokines (TNF-α and IL6) [9,10,11,12,13].

VD can be obtained through two main pathways: endogenous synthesis and dietary intake. Approximately 80% of the VD pool is derived from endogenous synthesis, where 7-dehydrocholecalciferol is converted to cholecalciferol (VD3) upon exposure to ultraviolet B (UVB) radiation in the skin. The remaining VD is obtained from dietary sources, mainly in the form of VD3. Rich dietary sources of VD3 include liver oil and fatty fish, followed by beef liver, dairy products, and egg yolks. The VD2 isoform is found in plant-based foods, with mushrooms being one of the few natural sources. This is particularly important for at risk populations such as the elderly, individuals with limited sun exposure, those with malabsorption disorders, athletes requiring enhanced recovery and immune support, and certain chronic conditions [5,14,15,16,17,18,19,20]. Some studies suggest that VD deficiency might contribute to chronic diseases associated with increased inflammation and immune system deregulation.

The fortification of foods with VD has become a widely adopted strategy to address deficiencies, considering that VD deficiency is a global public health concern [21]. Fortified products, including dairy items like milk and yogurt, plant-based beverages, orange juice, and cereals, have demonstrated efficacy in improving VD status and associated health outcomes, such as enhanced bone mineral density, cardiovascular risk markers, and immune function [14,22]. Additionally, there is growing interest in developing functional foods using protein hydrolysates combined with other vitamins, such as vitamin C and vitamin E, to enhance their bioactive properties. These products are particularly interesting for their potential to improve the bioavailability of these nutrients and to provide synergistic health benefits, especially in reducing oxidative stress and inflammation, providing new advancements and directions for functional foods [23].

On the other hand, enzymatic protein hydrolysates, consisting of a mixture of peptides and free amino acids, enhance their bioactive properties, in contrast with the non-hydrolyzed protein [24]. Chickpea protein hydrolysates, in particular, have shown an effective modulation of oxidative stress and inflammation in several studies [25,26,27,28,29]. These studies show their biological relevance not only in cellular models but also using molecular docking studies [30,31]. In a previous study, our group showed the physicochemical and antioxidant properties of a chickpea protein hydrolysate, H30BIO, through ex vivo assays [26]. Moreover, H30BIO exerts notable effects at the cellular level, suggesting mitochondrial protection and anti-inflammatory activity [32]. A few studies have also investigated the activity of chickpea protein hydrolysates in cellular lines, demonstrating an antioxidant capacity and immunomodulatory and antidiabetic activities [25,28,33,34].

Given the crucial role of VD in modulating immune responses, combining it with the chickpea protein hydrolysate “H30BIO”, which possesses antioxidant and anti-inflammatory properties, could enhance its therapeutic potential [32]. Unlike other VD-fortified foods, the combination of H30BIO and VD could result in a synergistic effect, boosting the anti-inflammatory response of the body. In light of these considerations, this study aims to investigate the anti-inflammatory effects of a VD-enriched protein supplement in a model of LPS-induced low-grade inflammation using THP-1 cells. We hypothesize that the combination of VD with hydrolyzed chickpea protein will more effectively modulate the inflammatory response compared to VD alone, providing valuable insights into potential therapeutic strategies for managing inflammatory-related conditions.

## 2. Results

### 2.1. Effect of VD on Cell Viability

**Figure 1:**
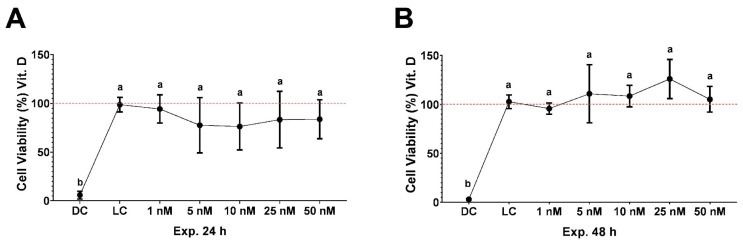
Cell viability analysis of THP-1 cells treated with different compounds at 24 and 48 h. The graph shows the 24 h viability of THP-1 cells treated with various concentrations of VD (1–50 nM) (**A**) and 48 h viability at the same concentrations for VD (**B**). Data are expressed as the mean (percentage of absorbance compared with that obtained in the control (non-treated cells)) ± SD, and different letters (a,b) were assigned in the multiple comparison test across all groups, indicating significant differences in a *p* value < 0.05, *n* ≥ 4. DC corresponds to the dead cells control and LC to the live cells control.

An MTT assay was employed to evaluate the viability or cytotoxicity of VD (Figure 1). This assay relies on the oxidative metabolism of cells, particularly the mitochondrial production of ATP [35]. The data revealed that after 24 h (Figure 1A) and 48 h (Figure 1B) of exposure to VD, there were no significant differences in cell viability compared to the live control. The viability study of H30BIO demonstrated that it is a non-toxic ingredient, as described in detail by Rodríguez-Martin et al., 2022 [32].

### 2.2. The Effect of VD on Pro-Inflammatory Cytokines and Genes

**Figure 2:**
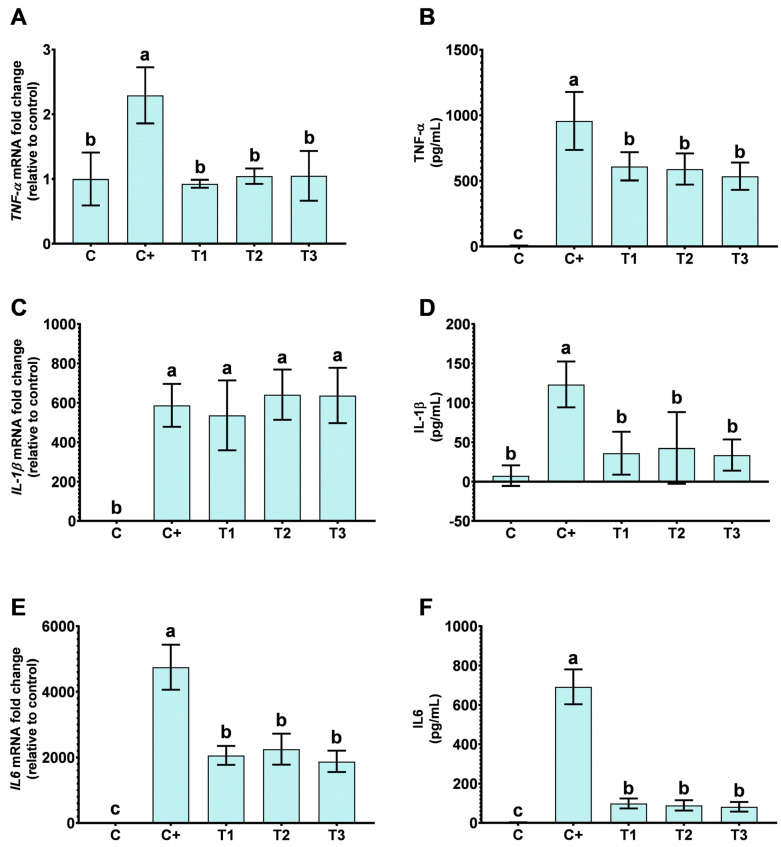
Pro-inflammatory cytokines and genes in LPS-induced inflammation THP-1 cells treated with VD. The figure shows the mRNA of *Tumor Necrotic Factor-α* (*TNF-α*) levels (**A**), as well as the content of TNF-α in the supernatant of the treated cells described below (**B**). In (**C**), the figure shows the expression levels of *Interleukin-1β* (*IL-1β*) and the release of the IL-1β in cellular supernatants after the treatments (**D**). (**E**,**F**) show mRNA levels and content in the supernatant of Interleukin 6 (IL6), respectively. Data are expressed as the mean ± SD, and different letters (a–c) indicate statistical differences in the multiple comparison test across all groups using a *p* value < 0.05, *n* = 6. Treatments conducted were C: without reactive; C+: Lipopolysaccharide (LPS) (50 ng/mL); T1: LPS (50 ng/mL) + vitamin D (VD) (10 nM); T2: LPS (50 ng/mL) + VD (25 nM); T3: LPS (50 ng/mL) + VD (50 nM).

The release of pro-inflammatory cytokines TNF-α, IL-1β, and IL6 into the cellular medium was quantified to evaluate the impact of VD treatment. As illustrated in Figure 2B,D,F, a significant reduction in the levels of TNF-α, IL-1β, and IL6, respectively, was observed when cells were treated with VD at any concentration (T1, T2, T3) compared to the pro-inflammatory control (C+, low LPS concentration). Gene expression analysis (Figure 2A: TNF-α, Figure 2C: IL-1β, Figure 2E: IL6) revealed a substantial downregulation of TNF-α and IL6 expression following VD treatment relative to the LPS control (C+). However, IL-1β gene expression remained largely unchanged under the same conditions. Notably, no dose-dependent effect was detected across the VD-treated groups.

### 2.3. Effect of VD-Supplemented H30BIO on Pro-Inflammatory Cytokines and Genes

**Figure 3:**
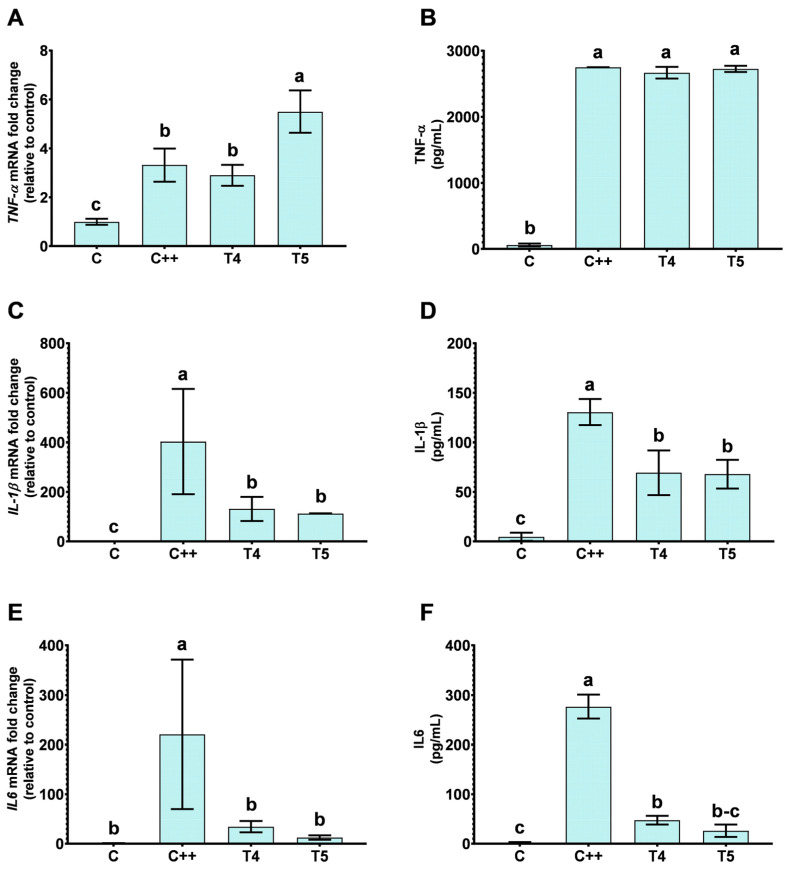
Pro-inflammatory cytokines and genes in LPS-induced inflammation THP-1 cells treated with H30BIO, alone and supplemented with vitamin D. The figure shows the mRNA of *Tumor Necrotic Factor-α* (*TNF-α*) levels (**A**), as well as the content of TNF-α in the supernatant of the treated cells described below (**B**). In (**C**), the figure shows the expression levels of *Interleukin-1β* (*IL-1β*) and the release of the IL-1β in cellular supernatants after the treatments (**D**). (**E**,**F**) show mRNA levels and content in the supernatant of *Interleukin-6 (IL-6)*, respectively. Data are expressed as the mean ± SD, and different letters (a–c) indicates statistical differences in the multiple comparison test across all groups using a *p* value < 0.05, *n* = 6. Treatments conducted were C: without reactive; C++: Lipopolysaccharide (LPS) (100 ng/mL); T4: LPS (100 ng/mL) + H30BIO (250 µg/mL); T5: LPS (100 ng/mL) + vitamin D (10 nM) + H30BIO (250 µg/mL).

Treatment with H30BIO (T4) and VD-supplemented H30BIO (T5) resulted in a significant reduction in the release of IL-1β (Figure 3D) and IL6 (Figure 3F) in the cellular supernatants compared to the control, which was further confirmed by the downregulation of their gene expression, as shown in Figure 3C and Figure 3E, respectively. There were no differences between the effects of H30BIO alone and VD-supplemented H30BIO. Interestingly, the release of TNF-α (Figure 3B) remained comparable to that of the LPS control (C++) with both treatments. However, the gene expression of TNF-α (Figure 3A) was similar to C++ in cells treated with H30BIO alone but significantly elevated in those treated with VD-supplemented H30BIO.

### 2.4. Effect of VD and VD-Supplemented H30BIO on NF-κB Pathway Genes

**Figure 4:**
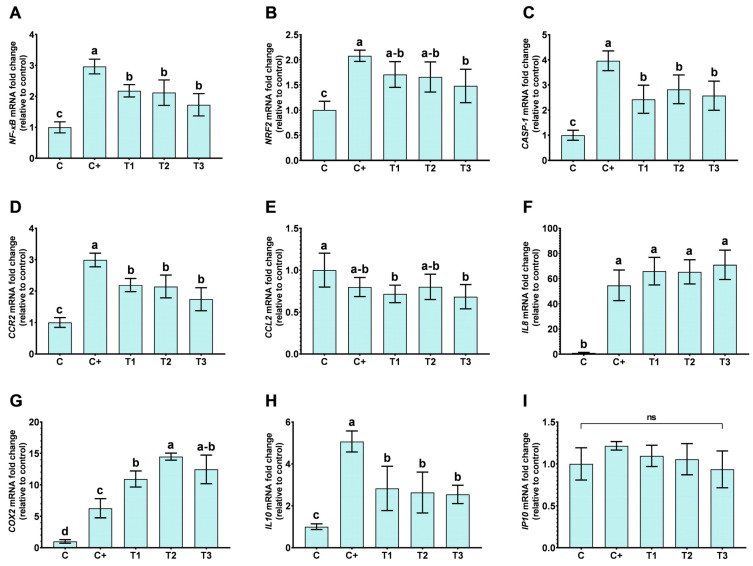
Gene transcription levels of NF-κB pathway in LPS-induced inflammation THP-1 cells treated with vitamin D. The figure shows the relative levels of gene expression of *Nuclear Factor Kappa B Subunit 1* (*NF-κB*) (**A**), *Nuclear Factor Erythroid 2-related Factor 2* (*NRF2*) (**B**), *Caspase-1* (*CASP-1*) (**C**), *C-C Motif Chemokine Receptor 2* (*CCR2*) (**D**), *C-C Motif Chemokine Ligand 2* (*CCL2*) (**E**), *Interleukin 8* (*IL8*) (**F**), *Cyclooxygenase-2* (*COX2*) (**G**), *Interleukin 10* (*IL10*) (**H**), and *C-X-C motif chemokine ligand 10* (*CXCL10*, *IP10*) (**I**). Data are expressed as the mean ± SD, and different letters (a–c) were assigned in the multiple comparison test across all groups, indicating significant differences at a *p* value < 0.05; and ‘ns’ indicates non-statistical differences in the multiple comparison test, *n* = 6. Treatments conducted were C: without reactive; C+: Lipopolysaccharide (LPS) (50 ng/mL); T1: LPS (50 ng/mL) + vitamin D (VD) (10 nM); T2: LPS (50 ng/mL) + VD (25 nM); T3: LPS (50 ng/mL) + VD (50 nM).

Gene expression analyses were performed to investigate how VD influences the reduction in LPS-induced inflammation within the primary inflammatory pathway *NF-κB*. As shown in Figure 4, significant changes in most gene expressions were observed in VD treatments compared to the LPS control (C+, low LPS concentration). Notably, treatments with all concentrations of VD led to a significant decrease in the expression of the *NF-κB* and *CASP-1* genes. Additionally, a downregulation trend in *NRF2* expression was observed, which became significant at higher VD doses.

**Figure 5:**
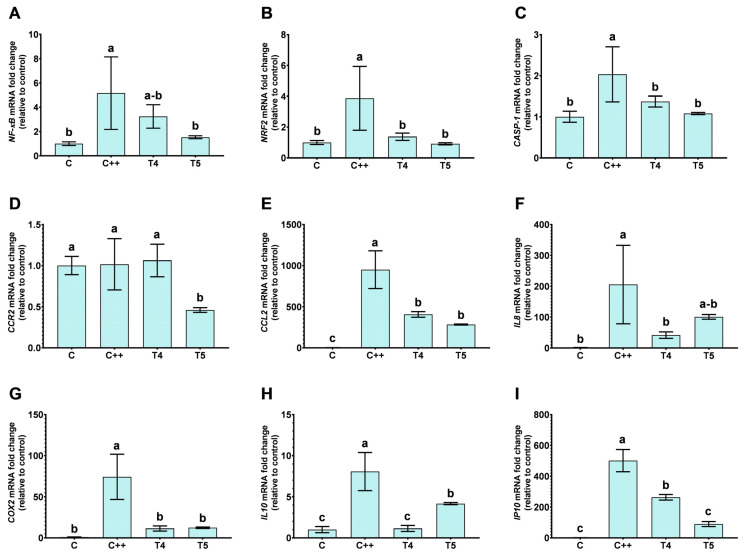
Gene transcription levels of NF-κB pathway in LPS-induced inflammation in THP-1 cells treated with H30BIO alone and supplemented with vitamin D. The figure shows the relative levels of gene expression of *Nuclear Factor Kappa B Subunit 1* (*NF-κB*) (**A**), *Nuclear Factor Erythroid 2-related Factor 2* (*NRF2*) (**B**), *Caspase-1* (*CASP-1*) (**C**), *C-C Motif Chemokine Receptor 2* (*CCR2*) (**D**), *C-C Motif Chemokine Ligand 2* (*CCL2*) (**E**), *Interleukin 8* (*IL8*) (**F**), *Cyclooxygenase-2* (*COX2*) (**G**), *Interleukin 10* (*IL10*) (**H**), and *C-X-C motif chemokine ligand 10* (*CXCL10*, *IP10*) (**I**). Data are expressed as the mean ± SD, and different letters (a–c) were assigned in the multiple comparison test across all groups, indicating significant differences at a *p* value < 0.05, *n* = 6. Treatments conducted were C: without reactive; C++: Lipopolysaccharide (LPS) (100 ng/mL); T4: LPS (100 ng/mL) + H30BIO (250 µg/mL); T5: LPS (100 ng/mL) + VD (10 nM) + H30BIO (250 µg/mL).

Parallel experiments with H30BIO (T4) and VD-supplemented H30BIO (T5) (Figure 5) demonstrated that H30BIO alone and supplemented with VD downregulated *NRF2* and *CASP-1* compared to the control. Notably, the combination of VD and H30BIO exhibited a significant effect on the downregulation of *NF-kB* compared to H30BIO alone.

### 2.5. Effect of VD and VD-Supplemented H30BIO on M1/M2-Related Markers

**Figure 6:**
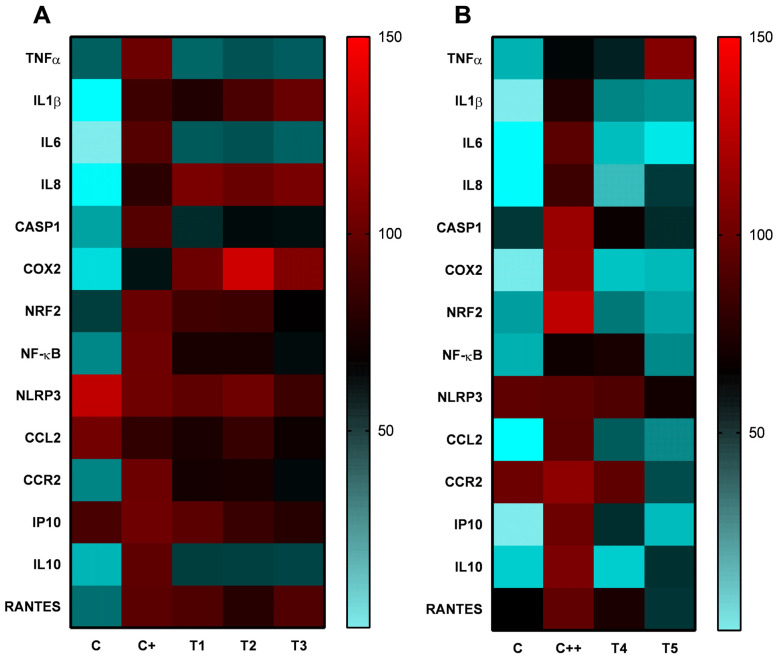
Heat map of gene expression in LPS-induced inflammation THP-1 cells treated with vitamin D and/or H30BIO. The heat map represents gene expression normalized to the overstimulated control (C+ (**A**) or C++ (**B**)), with a color scale ranging from 0% (minimal expression, blue) to 150% (expression 50% higher than the C+ or C++ controls, red), *n* = 6. More intense red indicates higher levels of relative expression, while more light blue indicates lower levels. Treatments conducted were C: without reactive; C+: Lipopolysaccharide (LPS) (50 ng/mL); T1: LPS (50 ng/mL) + VD (10 nM); T2: LPS (50 ng/mL) + VD (25 nM); T3: LPS (50 ng/mL) + vitamin D (VD) (50 nM); C++: Lipopolysaccharide (LPS) (100 ng/mL); T4: LPS (100 ng/mL) + H30BIO (250 µg/mL); T5: LPS (100 ng/mL) + VD (10 nM) + H30BIO (250 µg/mL).

In this study, the stimulation of undifferentiated THP-1 cells with LPS altered the expression of markers associated with monocytic activation. Although these cells were not differentiated into macrophages, the observed changes are similar to the subset macrophage profile, indicating a pro-inflammatory activation induced by LPS. In this regard, the responses of all genes in the different treatments were analyzed using a normalized approach (Figure 6). In relation to VD treatment, the analysis of polarization markers through gene expression showed that VD did not significantly alter the expression of *IL8* or *IP10*, as observed in Figure 4 and Figure 6A, while *COX2* expression was upregulated following VD treatment. Treatment with H30BIO alone resulted in the downregulation of *IL8*, *IP10*, *IL10*, *CCL2*, and *COX2*, while *CCR2* mRNA expression levels remained unchanged (Figure 5 and Figure 6B). In VD-supplemented H30BIO treatments, there was a downregulation of *CCR2* and *CCL2* compared to the LPS control. Interestingly, the downregulation of *IL10* and *IL8* was also observed in this case, but these expressions were higher in comparison with the H30BIO treatment alone; additionally, the IP10 downregulation was higher using both compounds (Figure 5 and Figure 6B).

When cells were treated with VD-supplemented H30BIO, the mRNA levels of *IP10*, *CCR2*, and *CCL2* were lower compared to the treatment with H30BIO alone. *COX2* expression levels were similar between the VD-supplemented H30BIO and H30BIO treatments. Additionally, *IL10* and *IL8* expression levels were slightly higher in the VD-supplemented H30BIO treatment compared to H30BIO alone.

### 2.6. Enrichment Analyses

**Figure 7:**
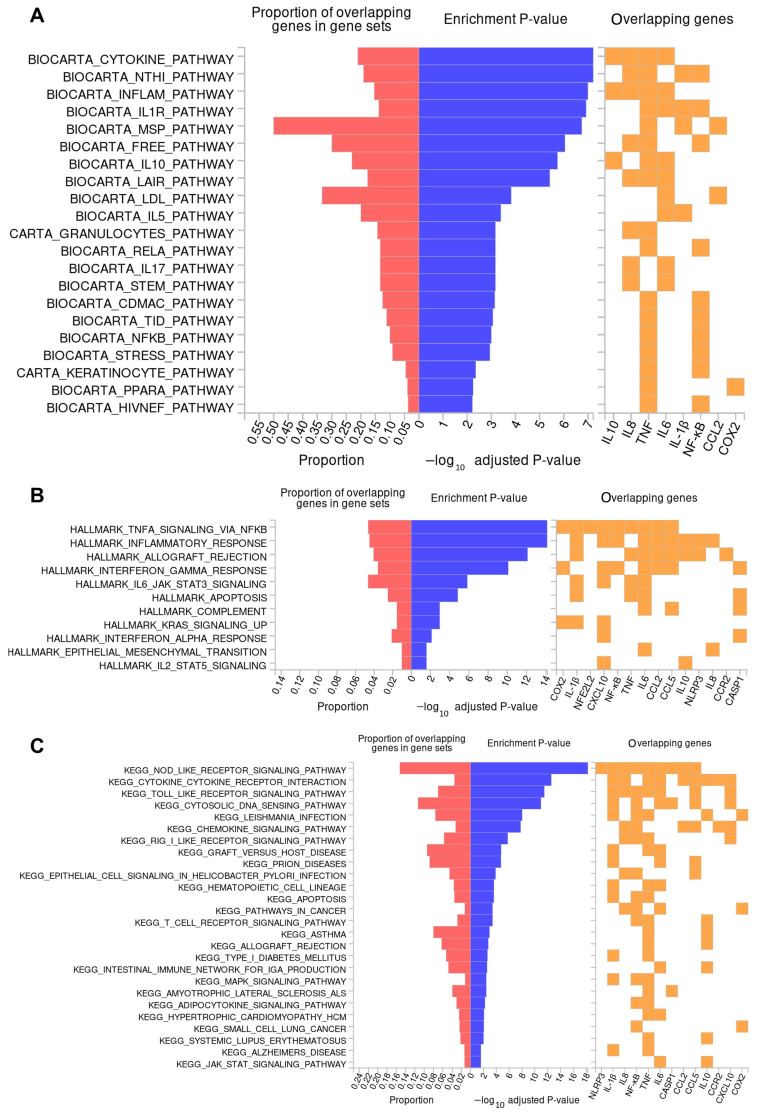
Enrichment analysis using Biocarta (**A**), Hallmark (**B**), and KEGG (**C**) databases. Enrichment adjusted log-transformed *p*-values and proportions of overlapping genes are displayed alongside corresponding pathway names. Key pathways include cytokine signaling, NF-κB activation, inflammatory response, and apoptosis. The results provide insights into the molecular and cellular processes associated with the analyzed gene set.

The enrichment analysis results presented in Figure 7 highlight several key pathways associated with inflammation and immune response processes. Particularly, Figure 7A shows results derived from the Biocarta, 7B Hallmark, and 7C KEGG databases.

These pathways show a significant overrepresentation of the genes involved in cytokine-cytokine receptor interactions, including *IL-1β*, *IL8*, *TNF-α*, *IL6*, *CCL2*, *RANTES (CCL5)*, *IL10*, *CCR2*, and *IP10*. These cytokines are crucial mediators of inflammation, and the analysis underscores their role in regulating immune cell activation, with the *NF-κB*, cytokine, and NOD-like receptor pathways further driving these processes through *IL6*, *IL8*, *IL10*, and *TNF*-α expression. The involvement of these genes points to their significance in modulating immune responses, particularly in inflammation-driven conditions.

Additionally, the analysis identifies genes linked to the activation and signaling of immune cells such as macrophages, neutrophils, and natural killer (NK) cells (*IL-1β*, *TNF-α*, *IL8*), which are essential in the body’s response to infections and tissue damage. The NF-κB pathway, a pivotal regulator of inflammation, also shows enrichment with regard to the majority of the genes, playing a major role in controlling immune responses and the expression of inflammatory mediators (Figure 7B). Importantly, the analysis suggests that these enriched pathways are associated with NOD-like and cytokine receptor signaling-related diseases, such as autoimmune conditions including asthma, rheumatoid arthritis, and systemic lupus erythematosus, amongst others, emphasizing how dysregulated inflammation may contribute to these diseases (Figure 7C). The results suggest that the enriched protein hydrolysate may be valuable for interacting with these pathways.

## 3. Discussion

Low-grade and systemic chronic inflammation can arise due to various factors, including increased body fat, sleep, and stress, among others linked to poor habits, environmental influences, or genetic predisposition. In addition, chronic or infectious diseases or microbiome dysbiosis contribute to the exacerbation of chronic inflammation, and it often escalates with age. Low-grade inflammation is associated with an increased risk of developing several non-communicable diseases, such as cancer, cardiovascular diseases, type 2 diabetes, depression, and neurodegenerative and autoimmune disorders [36]. Given that approximately 74% of global deaths are attributable to non-communicable diseases [37], it is of interest to design dietary strategies to control many of the mentioned factors. The study of functional ingredients capable of managing inflammation is an emerging means to improve inflammation status, human health, and longevity.

In this study, we used THP-1, a human monocytic leukemia cell line, which is commonly extended to study monocyte and macrophage functions, inflammatory mechanisms, signaling pathways, and nutrient and drug transport [38]. In this context, LPS is often used to induce inflammation in THP-1 cells, serving as a pro-inflammatory model [39,40,41]. Given the extensive acceptance and use of this model, investigating the synergistic actions between VD and the chickpea protein hydrolysate “H30BIO” on pro-inflammatory genes presents a valuable in vitro approach.

Inflammation is a cellular defense mechanism, which involves several immune cells, blood vessels, and pro-inflammatory molecules in an effort to remove cellular damage and to start the healing process in tissues. Signaling events, including the activation of transcriptional factor NF-κB, are triggered in this process. NF-κB maintains the control of the production and release of pro-inflammatory cytokines such as IL-1β, TNFα, and IL6 [42]. Otherwise, intracellular receptors such as the NLRP3 inflammasome and CASP-1 are part of an indispensable complex system responsible not only for the maturation and release of IL-1β but also for NF-κB positive feedback and reactive oxygen species production. VD may modulate these pathways, potentially affecting IL-1β secretion through post-transcriptional mechanisms, without altering gene expression [43]. Additionally, other chemokines act as recruiting immune cells, such as IL8 and CCL2 [44]. Together, these elements create a cytokine storm involved in the pro-inflammatory response but also maintain a special role in the inflammation’s resolution. These molecules, among others, can act in negative or positive feedback, which is critical for understanding the resolution phase that allows for the return to cellular homeostasis and the healing processes. And several signaling molecules act as activating or deactivating proteins and molecules, serving as an enhancer in this process.

Our study demonstrated that VD, both alone and in combination with the protein hydrolysate H30BIO, significantly modulates the expression of key pro-inflammatory cytokines and genes involved in the NF-κB pathway. Specifically, VD treatment led to a marked reduction in the expression of *TNF-α* and *IL6*, as well as a significant downregulation of the *NF-κB* and *CASP-1* genes, suggesting a potent anti-inflammatory effect. In line with these results, in vitro assays show that VD3 promotes dendritic cell differentiation, characterized by the presence of low levels of inflammatory cytokines such as *TNF-α* and high levels of *IL10*, as an anti-inflammatory cytokine [9,10]. Moreover, other studies show that the active form of VD inhibits the LPS-induced production of *IL6* and *TNF-α* by human monocytes in a dose-dependent manner, supporting the role of VD as an immunomodulatory agent and corroborating our results [11,12,13].

Interestingly, while H30BIO alone reduced pro-inflammatory markers (*IL-1β*, *IL6*) and did not maintain action through *TNF-α*, also contrasting with previous results ([32], article under review), its combination with VD resulted in a downregulation of pro-inflammatory genes in the same manner as H30BIO (while creating an upregulation of *TNF-α* and also a downregulation of *IL10* in comparison with LPS) but higher than H30BIO alone, and a significative downregulation of *IP10*, indicating a synergistic effect not previously reported. In this context, TNF-α plays a particular role in the positive feedback of cytokine signaling pathways and also in healing processes [45]. Thus, we may hypothesize that the function of TNF-α depends on the activation of the soluble or membrane form of its receptors, tumor necrosis factor-alpha receptor 1 (TNFR-1) or 2 (TNFR-2), which can stimulate different signaling pathways. For instance, in monocyte cells, TNFR-1 is mainly related to pro-inflammatory behavior, while TNFR-2 can induce the downregulation of *IL8*, *IL1-β*, and *IL6*, as well as the 50% downregulation of *IL10* genes [46]. As Figure 5 and Figure 6 show, VD-H30BIO was implicated in the downregulation of *IL8*, *IL-1β*, *IL6* and the downregulation of *IL10*, but with a higher expression if compared with the H30BIO alone; thus, both compounds might interact with TNFR, driving TNF-α coupling to TNFR-2 instead of TNFR-1. In addition, some authors indicate that the major expression of TNFR-1 or 2 is related to different monocyte subsets. Non-classical monocytes expressed the highest levels of TNFR-2, and the highest expression of TNFR-1 was found on intermediates [47].

Regarding monocyte subsets, human monocytes are generally classified into three subsets, classical (CD14^++^ CD16^−^), intermediate (CD14^++^ CD16^+^), and non-classical (CD14^+^ CD16^++^), based on the expression of CD14 and CD16 markers [48,49,50]. However, since monocytes are key cells in the pathophysiological response to various stimuli, an increasing number of markers are being used to subdivide them into distinct functional subsets. The specific roles of these monocyte subsets in health and disease are still under investigation. In this context, other genes are considered in the clustering of monocyte differentiation, which is the case with *TNFR-1* and *2*, among others. For instance, the cytokine production of IL10, CCL2, IL8, or IL6 is associated with the classical subset; TNF-α, IL-1β, and IL6 are markers for the intermediate subset; and IL-1R, TNF-α and IL-1β characterize non-classical monocytes [50]. Moreover, Schmidl et al. (2014) identified that the motif signature of intermediate monocytes was dominated by the modulation of *NF-κB*. Additionally, their analysis suggests underlying epigenetic regulatory mechanisms among the three subsets, which are regulated by different metabolic processes—the glycolytic pathway in classical monocytes and oxidative phosphorylation in non-classical monocytes [51].

Results obtained in our study indicated that LPS stimulation (positive control) leads to an upregulation of gene expression, driving the activation of classical monocytes. This is due to an increased expression and production of cytokines and chemokines such as TNF-α, IL-1β, IL6, IL8, IP10, CCL2, and RANTES, as well as transcription factor NF-κB [50]. These data also align with the pro-inflammatory response pathways observed in the enrichment analysis using the Biocarta, Hallmark, and KEGG databases, suggesting the involvement of NF-κB, cytokine, and NLRP pathway activation through the overlapping genes (Figure 7). On the other hand, subsequent treatment with VD downregulated the expression and release of *TNF-α* and *IL6* and caused a 50% downregulation of *IL10*, while upregulating the expression of *COX2*, leading to the non-classical monocyte subset, and did not maintain its effect through *IL8*, *IP10*, or *CCL2*. This suggests an anti-inflammatory profile that would possibly enhance the release of reactive oxygen species (ROS), which could be modulated by VD itself. In this sense, the differentiation of monocyte subsets and the anti-inflammatory activity of VD observed align with previous studies. For instance, Yong Zhang et al. (2012) demonstrated that VD reduces TNF-α and IL6 levels in a similar cellular model by inhibiting LPS-induced p38 activation and cytokine production in human monocytes, closely related to *NF-κB* pathway activation [11]. Their study showed a significant decrease in the CD14^+^ cells induced by LPS through treatment with VD, suggesting the conversion to non-classical monocytes or macrophages in peripheral blood mononuclear cells (PBMCs). While other studies did not report an increase in *COX2* after exposure to VD in monocytes or macrophages, there is evidence of increased PGE_2_ synthesis in keratinocytes via an upregulation of *COX2* expression, which may promote or attenuate cutaneous inflammation (as a dual role) [52], but also oxidative phosphorylation related to non-classical monocytes [52] may be regulated by *COX2* pathways. Additionally, it is possible that the increase in COX2 expression in our study reflects a context-dependent response, where VD might enhance COX2 activity to support the inflammatory process [53]. This could represent a compensatory mechanism or a more regulated response to inflammation, which may explain the observed uptrend in COX2 expression in the VD + LPS group compared to LPS alone.

Finally, the expression and release of cytokines and chemokines after combined treatment with VD and H30BIO indicated a synergistic effect on possible cell differentiation. This is evidenced by the upregulation of *TNF-α*, the 50% downregulation of *IL10*, and the downregulation of *COX2*, *IL8*, *RANTES*, and *CCL2*, along with the most significant downregulation of *NF-κB* and *IP10* observed. This pattern suggests differentiation towards a monocyte subset between the intermediate and non-classical monocytes [49]. In this context, an enrichment analysis using Biocarta indicated that the *IL10* and *TNF-α* genes are highly correlated with NF-κB and related pathways such as RELA (Transcription Factor P65) and the HIVNEF (HIV-1 Nef-Interacting Protein), which plays a role in autoimmunity through the regulation of *NF-κB* [54]. Furthermore, these cytokines are associated with the modulation of ATP production in the mitochondria through the TID (DnaJ Homolog Subfamily A Member 3, Mitochondrial) pathway [54]. On the other hand, other studies suggest that H30BIO shows a protective action on the mitochondria, restoring SOD2 (Superoxide Dismutase 2) at activity and gene expression levels [32].

Given the results obtained, the synergic anti-inflammatory activity of VD with H30BIO could have significant therapeutic potential in managing chronic and low-grade inflammatory conditions. The ability of VD-supplemented H30BIO to downregulate key inflammatory markers more effectively than VD alone suggests that this combination could enhance the efficacy of existing treatments in certain cases or serve as a novel therapy. Despite the promising results, further investigation is needed to fully understand the overall effect and to test the potential beneficial effect in in vivo studies.

## 4. Materials and Methods

### 4.1. Chemicals and Sampling

1α,25-Dihydroxyvitamin D3 was provided by Sigma Chemical Co. (St Louis, MO, USA, Cat. No. 32222-06-3). Lipopolysaccharide (LPS, *E. coli* 055: B5) was purchased from Sigma-Aldrich (St Louis, MO, USA; Ref: L2637). The chickpea protein hydrolysate H30BIO was obtained by the Group of Plant Protein at the Instituto de la Grasa-CSIC (Seville, Spain) [26]. Cell culture media and other cellular-grade reagents were purchased from Biowest (Nuaille, France) and Gibco, Thermo Fisher Scientific (Madrid, Spain).

3-(4,5-dimethylthiazol-2-yl)-2,5-diphenyltetrazolium bromide (MTT) was purchased from Sigma-Aldrich (St. Louis, MO, USA; Cat. No. 298-93-1). Trisure (Bioline, Meridian Life Science, Inc., Memphis, TN, USA) was used to isolate total RNA, and a synthesis kit from Bio-Rad (Hercules, CA, USA; Ref: 1708890) was used to reverse transcribe it to cDNA. Primers were designed using Primer3 software (open-source, developed by Helen J. Skaletsky and Steve Rozen at the Whitehead Institute, Cambridge, MA, USA; maintained by the Primer3 community, GitHub repository) and synthesized by Eurofins Genomics (Ebersberg, Germany). Real-Time Quantitative PCR was performed using the iTaq™ Universal SYBR^®^ Green Supermix of Bio-Rad (Hercules, CA, USA; Ref: 1725120).

All chemicals (reagents and solvents) were of biomolecular grade and were provided by Sigma-Aldrich (St. Louis, MO, USA), Bachem AG (Bubendorf, Switzerland), and Gibco, Thermo Fisher Scientific (Madrid, Spain). The disposable plastics used in the experiments were DNase and RNase-free, sterile, and were mainly supplied by Thermo Fisher Scientific (Madrid, Spain).

### 4.2. Cell Culture Maintenance

The THP-1 cells were obtained from the Cell Biology Unit at the Instituto de la Grasa (Seville, Spain). The cells were maintained in Roswell Park Memorial Institute (RPMI)-1640 medium, supplemented with 10% heat-inactivated fetal bovine serum (Biowest, Nuaillé, France) and 1% penicillin/streptomycin (Gibco, Thermo Fisher Scientific, Madrid, Spain). They were incubated in 5% CO2 at 37 °C in a CO_2_ incubator (Binder CB170L, Binder GmbH, Tuttlingen, Germany).

### 4.3. Cell Toxicity Assay

Cytotoxicity was measured using the MTT method. THP-1 cells were incubated in 96-well plates at a density of 1 × 10^6^ cells/mL for 24 h and 48 h with VD (1, 5, 10, 25, and 50 nM). In these plates, cell life (only live cells) and cell death (live cells with triton) controls were included. After this period, the MTT solution was added and incubated over 3 h until a purple precipitate was visible. MTT-formazan crystals were solubilized with DMSO (Sigma-Aldrich, St. Louis, MO, USA) and then measured with a microplate reader at 570 nm, corrected to 650 nm. Cell survival was expressed as the percentage of absorbance compared with that obtained in the control (non-treated cells).

### 4.4. Cellular Treatments

In this study, 50 ng/mL LPS was used to induce a moderate inflammatory response for VD treatment (at 10, 25, or 50 nM) to determine the optimal concentration of VD, while 100 ng/mL LPS was employed to generate a stronger inflammatory challenge for the combined treatment with VD (at 10 nM) and H30BIO (250 µg/mL).

In the first experiment, cells were treated with LPS and/or VD. This experiment maintained different conditions consisting of a negative control (non-LPS and non-VD added), positive control (cells stimulated with LPS at 50 ng/mL), and treatments in LPS-stimulated cells with VD at 10, 25, or 50 nM (T1, T2, T3). The LPS was added, and it was left to incubate for 1 h. Finally, VD was added in the selected concentrations and allowed to incubate for 24 h.

The second experiment included LPS at 100 ng/mL. Subsequently, after 1 h incubation, the chickpea protein hydrolysate (H30BIO) at a concentration of 250 µg/mL (T4) or the H30BIO with VD (10 nM) (T5) was added and incubated for 24 h. The concentrations used and experimental procedure for both experiments are indicated in Table 1.

Two different batches of experiments, in triplicate at least, were conducted.

### 4.5. Evaluation of Cytokine Secretion by ELISA

The cytokine release in the culture media collected after the treatments was determined by an ELISA kit (Diaclone, Besancon, France). The tumor necrosis factor alpha (TNF-α), Interleukin 1 Beta (IL-1β), and Interleukin 6 (IL6) concentrations were measured. All tests were carried out in strict accordance with the manufacturer’s instructions. ELISA data were calculated using a standard curve supplied in the commercial kit and expressed as pg/mL.

### 4.6. RNA Isolation and Real-Time Quantitative PCR Analysis

Treated THP-1 cells were harvested and total mRNA was isolated to quantify gene expression by qRT-PCR, using a CFX connect 96 system (Bio-Rad, Hercules, CA, USA). Briefly, total mRNA was extracted using the TRIsure reagent and alcohol-based extraction methods. mRNA quality was assessed by calculating the A260/A280 ratio using a NanoDrop ND-1000 spectrophotometer (Denovix, Wilmington, DE, USA, Model DS-11-FX). Isolated mRNA was subjected to reverse transcription using the iScript cDNA Synthesis Kit (Bio-Rad, Hercules, CA, USA). An amount of 10 ug of the resulting cDNA was used as a template for qRT-PCR amplifications with Brilliant SYBR green Supermix. The magnitude of change in mRNA expression for the candidate genes (Table 2) was calculated using the standard 2−(ΔΔCt) method. All data were normalized to the endogenous reference (*HPRT* and *GAPDH*) gene content and expressed as a relative fold change of the control. Table 2 shows the primer pairs of genes used in this work.

### 4.7. Statistical Analysis

All values are expressed as arithmetic means ± SD. Statistics were evaluated with Graph Pad Prism Version 8.0.1 software (GraphPad Software, San Diego, CA, USA). Differences between the sample groups were evaluated by a one-way analysis of variance (ANOVA), followed by Tukey’s multiple comparison test as a post hoc test. *p* values less than 0.05 were considered statistically significant.

A heat map was generated to visualize the relative expression of the genes analyzed by quantitative real-time PCR, using GraphPad Prism version 8.0.1 (GraphPad Software, San Diego, CA, USA). The expression data for each gene were normalized relative to the overstimulated control (C+ or C++), which was assigned a value of 100%. The heat map scale ranges from 0% to 150% relative to this control, allowing a visualization of expression levels both below and above the control.

### 4.8. Enrichment Analysis

We conducted enrichment analyses on the gene set selected in the previous steps using the FUMA platform’s GENE2FUNC module (FUMA, Vrije Universiteit Amsterdam, Amsterdam, The Netherlands; accessed via https://fuma.ctglab.nl/gene2func on 22 September 2024). This tool allowed us to gather information on gene expression patterns and shared molecular functions between the selected genes. Specifically, we obtained functional analysis results for molecular, cellular, and metabolic pathways. The platform also provides enrichment analysis results based on genes, pathways, and tissue-specific expression. It is integrated with several widely used resources, such as Gene Ontology (GO), WikiPathways, and the Molecular Signatures Database (MsigDB).

We focused primarily on the enrichment analysis conducted using this platform. For the enrichment analysis, hypergeometric tests were performed to assess whether our genes of interest were overrepresented in predefined gene sets. The background gene set, representing the “universe,” consisted of the remaining genes from our study. These predefined gene sets were compared against the background genes for pathway enrichment using multiple data repositories, including MsigDB (Broad Institute, Cambridge, MA, USA), WikiPathways (WikiPathways Project, Maastricht University, Maastricht, The Netherlands), GO (Gene Ontology Consortium, Sacramento, CA, USA), and KEGG (Kyoto Encyclopedia of Genes and Genomes, Kyoto University, Kyoto, Japan). GO classifies gene functions into three categories: cellular component, molecular function, and biological process. KEGG provides insights into metabolic pathways and annotations for the enzymes involved at each step. MsigDB includes canonical pathways from KEGG, Reactome (European Bioinformatics Institute, Hinxton, UK), WikiPathways, and other databases. All these resources were accessed on 22 September 2024.

## 5. Conclusions

Our study demonstrates the synergistic and complex interactions between VD and H30BIO in modulating pro-inflammatory gene expression in THP-1 cells within a low-grade inflammatory microenvironment. While VD downregulates *TNF-α*, H30BIO selectively causes a downregulation of *IL-1β* without affecting *TNF-α*. The combined treatment exerts a synergistic effect, significantly reducing the expression of *IL-1β*, *CCR2*, and *IP10*, while also increasing *TNF-α* expression and maintaining a 50% downregulation of *IL10* cytokine. These findings provide compelling evidence that VD, particularly when combined with H30BIO, exerts significant anti-inflammatory effects by modulating key cytokines and genes within the NF-κB pathway. This suggests the modulation of adaptive processes related to intermediate and non-classical monocyte subsets, as well as potential interactions with TNF receptors. Our results open new avenues for the development of combined nutraceuticals or functional ingredients aimed at more effectively controlling the low-grade inflammation involved in the origin of many chronic diseases.

## Figures and Tables

**Table 1:** Summary of cellular treatments during experimentation.

	C	C+	C++	T1	T2	T3	T4	T5
LPS (50 ng/mL)	**-**	**+**	**-**	**+**	**+**	**+**	**-**	**-**
LPS (100 ng/mL)	**-**	**-**	**+**	**-**	**-**	**-**	**+**	**+**
Vit. D (10 nM)	**-**	**-**	**-**	**+**	**-**	**-**	**-**	**+**
Vit. D (25 nM)	**-**	**-**	**-**	**-**	**+**	**-**	**-**	**-**
Vit. D (50 nM)	**-**	**-**	**-**	**-**	**-**	**+**	**-**	**-**
H30BIO (250 µg/mL)	**-**	**-**	**-**	**-**	**-**	**-**	**+**	**+**

C: control without reactive; C+ and C++: Lipopolysaccharide (LPS) (50 and 100 ng/mL, respectively); T1: LPS (50 ng/mL) + VD (10 nM); T2: LPS (50 ng(mL) + VD (25 nM); T3: LPS (50 ng/mL) + VD (50 nM); T4: LPS (100 ng/mL) + H30BIO (250 µg/mL); T5: LPS (100 ng/mL) + H30BIO (250 µg/mL) + VD (10 nM).

**Table 2:** Primer pairs of genes used in qRT-PCR experiments.

	Forward	Reverse	MN
*TNF-α*	TCCTTCAGACACCCTCAACC	AGGCCCCAGTTTGAATTCTT	NM_000594.3
*IL-1β*	CTGTCCTGCGTGTTGAAAGA	TTCTGCTTGAGAGGTGCTGA	NM_000576.2
*IL6*	TGCAATAACCACCCCTGACC	GCTACATTTGCCGAAGAGCC	NM_000600.5
*IL8*	AGGAAAACTGGGTGCAGAGG	CCCTACAACAGACCCACACA	NM_000584.4
*CASP-1*	CAAGACCTCTGACAGCACGT	GCAGGCCTGGATGATGATCA	NM_001257119.3
*COX2*	GGTGACATCGATGCTGTGGA	AGTGCTTGGCTTCCAGTAGG	NM_000963.4
*NRF2*	GCGACGGAAAGAGTATGAGC	GTTGGCAGATCCACTGGTTT	NM_006164.5
*NF-κB1*	CTACGATGGAACCACACCCC	GGTTCCAGGCACAACTCCTT	NM_003998.4
*NLRP3*	GGAGAGAGCTGCGATCCATC	ACAACACCCGATGCTGTCAT	NM_001127462.3
*CCL2*	CCCCAGTCACCTGCTGTTAT	TGGAATCCTGAACCCACTTC	NM_002982.3
*CCR2*	GTGTGTGGAGGTCCAGGAGT	AAGCCAGACGTGTGATTTCC	NM_001123041.2
*IP10 (CXCL10)*	GCAAGCCAATTTTGTCCACGT	GTGGTCCATCCTTGGAAGCA	NC_000004.12
*NRF2*	GCGACGGAAAGAGTATGAGC	GTTGGCAGATCCACTGGTTT	NM_006164.5
*COX2*	GGTGACATCGATGCTGTGGA	AGTGCTTGGCTTCCAGTAGG	NM_000963.4
*IL10*	TGCAAAACCAAACCACAAGA	TCTCGGAGATCTCGAAGCAT	NM_000572.2
*RANTES*	AGGATCAAGACAGCACGTGG	TACTCCTTGATGTGGGCACG	NM_002985.3
*GAPDH*	ACAGTCAGCCGCATCTTCTT	ACGACCAAATCCGTTGACTC	NM_001289745.2
*HPRT*	TGGCGTCGTGATTAGTGATGA	AGAGGGCTACAATGTGATGGC	NM_000194.3

## Data Availability

The original contributions presented in this study are included in the article. Further inquiries can be directed to the corresponding author.
